# Coping as a Pathway Linking Religiosity and Spirituality to Mental Health and Early Cardio-Cerebrovascular Risk Among University Students in Malaysia

**DOI:** 10.3390/ijerph23060738

**Published:** 2026-05-31

**Authors:** Zaw Myo Hein, Anastasiya Spaska, Abdullah Duraid Nasif Jasim, Hafizah Abdul Hamid, Usman Jaffer, Che Mohd Nasril Che Mohd Nassir

**Affiliations:** 1 Department of Basic Medical Sciences, College of Medicine, Ajman University, Ajman P.O. Box 346, United Arab Emirates; z.hein@ajman.ac.ae (Z.M.H.); anastasiaspaska@gmail.com (A.S.); abdullahnasifmed@gmail.com (A.D.N.J.); 2 Department of Human Anatomy, Faculty of Medicine and Health Sciences, Universiti Putra Malaysia, Serdang 43400, Selangor, Malaysia; a_hafizah@upm.edu.my; 3 Abdul Hamid Abu Sulayman Kulliyyah of Islamic Revealed Knowledge and Human Sciences, International Islamic University Malaysia, Kuala Lumpur 50728, Malaysia; 4 Department of Anatomy and Physiology, School of Basic Medical Sciences, Faculty of Medicine, Universiti Sultan Zainal Abidin, Kuala Terengganu 20400, Terengganu, Malaysia

**Keywords:** mental health, spirituality, religiosity, university student, cardio-cerebrovascular risk

## Abstract

**Highlights:**

**Public health relevance—How does this work relate to a public health issue?**

- Common mental health disorders (anxiety, depression, stress) are prevalent among students and are linked to elevated cerebrocardiovascular disease (CCVD) risk.
- Spirituality and religiosity shape coping behaviors, influencing stress responses and downstream cardiovascular vulnerability.

**Public health significance—Why is this work of significance to public health?**

- Identifies psychosocial and behavioral determinants of early CCVD risk in a high-risk, understudied young population.
- Suggests that coping style may modify the association between religiosity and mental health outcomes.

**Public health implications—What are the key implications or messages for practitioners, policymakers and/or researchers in public health?**

- Integrating mental health screening with lifestyle and CCVD risk assessment should be prioritized in student health programs.
- Interventions should promote adaptive (positive) coping strategies while addressing maladaptive religious coping to potentially reduce early indicators of CCVD vulnerability.

**Abstract:**

Background: Mental health disorders such as depression, anxiety, and stress are increasingly prevalent among university students and contribute to long-term cardio-cerebrovascular disease (CCVD) risk. However, limited research has examined the interplay between mental health, CCVD risk factors, and religiosity/spirituality within Southeast Asia’s multicultural context. Methods: This cross-sectional study investigated these relationships among 484 undergraduate students enrolled in medical and health sciences programs across Peninsular Malaysia. Mental health status was assessed using the Depression, Anxiety, and Stress Scale (DASS-21). Self-reported clinical indicators associated with early CCVD vulnerability were also assessed. Religiosity and spirituality were measured using the Duke University Religion Index (DUREL), Brief Religious Coping (RCOPE), Spirituality Scale (SS), and Spiritual Coping Questionnaire (SCQ). Results: High prevalence rates of severe anxiety (50.4%), depression (29.3%), and stress (21.1%) were observed, with significant associations across ethnicity, religion, and academic programs. Higher religiosity and spirituality were generally associated with better mental health outcomes. However, coping style emerged as a key modifier of the relationship between religiosity/spirituality and mental health outcomes, with negative religious coping associated with greater psychological distress, whereas positive coping demonstrated mixed associations and partial mediating effects. Students with poorer mental health also exhibited higher CCVD risk burden. Conclusions: These findings highlight the importance of culturally and spiritually sensitive strategies in promoting student well-being.

## 1. Introduction

Psychological distress among university students has emerged as an increasing global public health concern, with escalating prevalence of depression, anxiety, and stress documented across various higher education environments [1,2]. Students in medical and health sciences programs are particularly vulnerable due to their intense academic workloads, high performance expectations, and exposure to emotionally demanding clinical environments. In Malaysia, this issue is especially pressing; the National Health and Morbidity Survey 2023 reported that nearly 40% of university students experience psychological distress, with significant implications for academic performance, social functioning, and long-term health outcomes [3].

Beyond the immediate psychological toll, poor mental health is increasingly recognized as a modifiable risk factor for non-communicable diseases, particularly cardio-cerebrovascular disease (CCVD). Evidence suggests that chronic psychological stress, depression, and anxiety contribute to alterations in hypothalamic–pituitary–adrenal (HPA) axis activity and disruption of autonomic nervous system homeostasis, endothelial dysfunction, and systemic inflammation, all of which have been associated with elevated CCVD risk in prior studies [4,5]. Alarmingly, the prevalence of CCVD among young adults is on the rise globally, with early-life risk factors, such as sedentary behavior, smoking, hypertension, and psychological stress, gaining attention as key contributors [6,7]. In Malaysia, CCVD remains the leading cause of death, accounting for nearly one in four deaths nationwide, and early markers of risk are increasingly observed among young populations [8]. Rather than examining these factors in isolation, this study adopts a pathway-based approach, positing that religiosity and spirituality are associated with mental health outcomes through coping mechanisms, which may in turn relate to early indicators of CCVD risk.

Amid growing efforts to address student mental health, increasing attention has been directed toward the roles of religion and spirituality as psychosocial resources. Religion is commonly conceptualized as adherence to organized beliefs, practices, and rituals associated with institutional traditions [9], whereas spirituality is typically understood as a more personal and subjective search for meaning, purpose, and connection with the transcendent [10]. Although these constructs are conceptually distinct, they are often overlapping and context-dependent, particularly in multicultural societies where religious identity and spiritual experience are deeply intertwined [11,12]. Both constructs have been associated with improved coping, greater psychological resilience, and enhanced emotional well-being [13,14]. However, their effects are multifaceted: positive religious and spiritual coping strategies, such as seeking comfort through faith or prayer, are typically linked to reduced psychological distress, while negative coping strategies, including spiritual discontent or perceiving adversity as divine punishment, may exacerbate mental health issues [15,16].

In a multicultural and multireligious society like Malaysia, home to Islam, Buddhism, Christianity, Hinduism, and traditional Chinese beliefs, religious and spiritual dimensions are deeply embedded in daily life and personal identity [8,17]. Understanding how these dimensions influence mental health and associated physical health risks is essential for designing culturally sensitive and effective interventions. Yet, most existing research on religiosity and mental health originates from Western contexts, with limited focus on Southeast Asian settings [18,19].

Emerging Malaysian studies suggest that spiritual practices may offer protective benefits. For example, Wong et al. [20] demonstrated that students who consistently participated in spiritual practices exhibited reduced levels of depression and anxiety. Likewise, Musa et al. [21] reported that the use of spiritual coping strategies was linked to improved psychological adaptation. However, few studies have comprehensively explored how both positive and negative forms of religious and spiritual coping interact with mental health and CCVD risk factors among university students, especially those in high-stress disciplines like medicine and health sciences. Despite increasing attention to religion, spirituality, and student mental health, there remains a notable gap in understanding how these factors interact with early physical health indicators, particularly among university students in Southeast Asia.

To address this gap, the present study is guided by the premise that religiosity and spirituality are associated with psychological well-being through distinct coping mechanisms, which may in turn be associated with early indicators of cardio-cerebrovascular disease (CCVD) risk. Specifically, we hypothesize that higher levels of religiosity and spirituality are associated with lower levels of depression, anxiety, and stress; that positive religious and spiritual coping strategies mediate this protective relationship, whereas negative coping strategies exacerbate psychological distress; and that poorer mental health is associated with a greater burden of early CCVD risk factors. Accordingly, this study aims to examine the relationships between religiosity, spirituality, mental health, and coping styles in a cohort of medical and health sciences students, while also exploring their associations with socio-demographic factors and early CCVD risk indicators. By integrating these dimensions, this research seeks to provide a nuanced, culturally grounded understanding of the interplay between mental and physical health in a vulnerable student population, and to inform the development of holistic, preventive, and culturally responsive strategies to support student well-being and reduce long-term cardiovascular risk.

## 2. Materials and Methods

### 2.1. Study Design

This cross-sectional study was conducted to comprehensively examine the interrelationships among sociodemographic characteristics, mental health status, religiosity, and spirituality among medical and health sciences students. Using a combination of self-administered questionnaires, the study sought to provide a detailed understanding of how these variables interact within the university student population across Peninsular Malaysia.

### 2.2. Study Population

The study population comprised undergraduate students enrolled in medical and health sciences programs at universities across Peninsular Malaysia. Inclusion criteria included undergraduate students aged 18–25 years who were currently enrolled and actively attending courses and who provided informed consent. No exclusion criteria were applied based on self-reported history of neurological or psychiatric disorders, substance use, or other health conditions, as the study aimed to capture real-world variability in mental health and related risk factors within the student population. Participants were excluded only if they provided incomplete responses or did not meet the age or enrollment criteria. Information on clinical conditions, including mental health diagnoses and medication use, was collected through a self-reported clinical proforma and included in the analysis as relevant variables. However, participants were instructed to complete the questionnaire based on their current mental and physical state, and no formal diagnostic verification was performed.

### 2.3. Sample Size Determination

Sample size estimation was performed using G*Power version 3.1.9.7 for a multiple linear regression analysis (fixed model, R^2^ deviation from zero, one-tailed). The calculation was based on a small effect size (f^2^ = 0.02), considered suitable for identifying modest associations in psychosocial and behavioral research. Assuming a significance level (α) of 0.05, statistical power (1 − β) of 0.95, and five predictors in the regression model, the minimum required sample size was determined to be 543 participants. To account for potential non-responses or data exclusion, we added a 10% buffer, targeting an adjusted sample size of approximately 598 students. A purposive sampling approach was used to recruit participants from various medical and health sciences programs. Ultimately, 488 students consented and responded, with 484 complete and usable datasets included in the final analysis after excluding four incomplete responses.

Although the target sample size was not fully achieved, the final analytic sample of 484 participants remains adequate for multivariate regression and mediation analyses involving continuous outcomes. Nevertheless, the reduced sample size may limit statistical power for detecting small effect sizes and may affect the stability of estimates in subgroup analyses, particularly where category counts are low. These limitations have been considered in the interpretation of the findings, with emphasis placed on effect sizes and overall patterns rather than isolated statistical significance.

### 2.4. Data Collection Instruments and Procedures

Participants were enrolled through institutional announcements and electronic invitation notices distributed via email. Prior to participation, all respondents provided informed consent. Authorization to utilize the respective questionnaires and assessment instruments was granted by the original developers. Furthermore, the instruments were converted into digital format, and selected wording was culturally adapted to enhance suitability and contextual relevance for the Malaysian population. While validated Malay versions were used where available, certain instruments (e.g., Brief RCOPE) required contextual linguistic adaptation at the time of the study. Newly developed culturally specific instruments, such as the Brief RCOPE-Malay [22], were published after data collection and represent important tools for future research.

Data collection was conducted electronically through Google Forms. The survey platform was configured to ensure that participation was restricted to individuals who fulfilled the inclusion and exclusion criteria, had reviewed and understood the informed consent and study instructions, and voluntarily agreed to continue with the study. Before full-scale data collection, the adapted instruments were subjected to a brief pilot test to assess cultural and linguistic appropriateness in the Malaysian context.

A panel of experts in clinical psychology and public health evaluated the instruments for face validity and contextual relevance. Additionally, a pilot test involving 30 undergraduate students was conducted to assess clarity, comprehension, and ease of completion. Internal consistency reliability was examined for each instrument using Cronbach’s alpha on the current sample. All scales demonstrated high reliability: DASS-21: α = 0.89 (total scale); DUREL: α = 0.82; Brief RCOPE: P-RCOPE α = 0.91; N-RCOPE α = 0.86; Spirituality Scale: α = 0.93 (total scale); SCQ: P-SCQ α = 0.90; N-SCQ α = 0.85. These values confirm that the instruments maintained good internal consistency when applied to our Malaysian student sample.

### 2.5. Socio-Demographic and Clinical Proforma Data

This self-administered questionnaire was used to obtain information on age, sex, socioeconomic status, family background, and other pertinent sociodemographic characteristics. The psychometric scale used in this study was consulted using previous research [23,24,25]. In this study, CCVD-related outcomes were operationalized as self-reported clinical and familial risk indicators associated with cardio-cerebrovascular vulnerability, including diabetes, chronic kidney disease, hypertension treatment, severe mental illness, smoking status, and family history of heart disease. These variables were exploratory proxies of early health vulnerability rather than validated measures of absolute cardiovascular risk.

### 2.6. Depression, Anxiety, and Stress- 21 (DASS-21) Assessment

The DASS-21 was employed to evaluate the participants’ levels of depression, anxiety, and stress (Supplementary File S1). The instrument comprises three self-report subscales specifically developed to assess the emotional domains of depression, anxiety, and stress, with each subscale containing seven items of related content. The depression domain evaluates features such as dysphoria, hopelessness, diminished life satisfaction, self-criticism, reduced interest or involvement, anhedonia, and low motivation. The anxiety domain measures autonomic activation, skeletal muscle-related symptoms, situational anxiety, and subjective experiences of anxious affect. Meanwhile, the stress domain assesses persistent nonspecific arousal, including difficulty relaxing, heightened nervous tension, irritability, over-reactivity, agitation, and impatience. Subscale scores were derived by summing the responses corresponding to each respective domain.

The DASS-21 is grounded in a dimensional framework of psychological distress rather than a categorical diagnostic approach. Its development was based on the premise, subsequently supported by empirical evidence, that depression, anxiety, and stress observed in non-clinical and clinical populations differ primarily in severity rather than in kind. Consequently, the DASS-21 is intended to quantify the extent of emotional distress and does not serve as a diagnostic instrument for classification according to categorical systems such as the DSM or ICD.

### 2.7. Religiosity Assessment

Religiosity among students was assessed using the DUREL [26] (Supplementary File S2). The DUREL is a five-item instrument designed to evaluate three dimensions of religious involvement: organized religious activity (ORA), non-organized religious activity (NORA), and intrinsic religiosity (IR) [26]. ORA encompasses participation in collective religious practices, such as attendance at places of worship and involvement in religious gatherings. In contrast, NORA reflects privately practiced religious behaviors, including personal prayer and individual scripture reading. The IR component evaluates the extent of internalized religious commitment and personal religious motivation.

The DUREL has demonstrated strong psychometric properties, including high test–retest reliability (*r* = 0.91), satisfactory internal consistency (α = 0.78–0.91), and substantial convergent validity with other religiosity measures (*r* = 0.71–0.86) [27]. For application within the Malaysian setting, the instrument was translated into Malay as the DUREL-M (Duke Religious Index Malay Version) and subsequently validated among the Malaysian population. The Malay version showed good internal reliability, with a Cronbach’s alpha coefficient of 0.80 [28]. In this version, wording was culturally adapted for the Malaysian Muslim-majority context (e.g., “church or other religious meetings” → “mosque/surau or other religious gatherings”). The English sample items presented here are provided solely for clarity and do not represent the exact Malay phrasing used. It scores between a range of 5–27. A sample of items is:

No. 1 (ORA): “How often do you attend church or other religious meetings?”, scaled-answer: 1—Never; 2—Once a year or less; 3—A few times a year; 4—A few times a month; 5—Once a week; 6—More than once/week.

No. 4 (IR): “My religious beliefs are what really lie behind my whole approach to life”, scaled-answer: 1—Definitely not true; 2—Tends not to be true; 3—Unsure; 4—Tends to be true; 5—Definitely true of me.

### 2.8. Religious Coping Assessment

Religious coping among students was evaluated using the Brief Religious Coping Scale (Brief RCOPE) developed by Pargament et al. [15] (Supplementary File S3). The instrument comprises 14 items, including seven items assessing positive religious coping (P-RCOPE) and seven items measuring negative religious coping (N-RCOPE), to examine the role of religion in managing life stressors and challenges. Positive religious coping reflects the use of adaptive spiritual strategies, including maintaining a constructive relationship with God through prayer, meditation, and spiritual reflection during periods of distress. In contrast, negative religious coping involves maladaptive interpretations of adversity, such as perceiving difficulties as divine punishment or attributing misfortune to abandonment or blame by God [15].

The Brief RCOPE has demonstrated strong psychometric performance, with median internal consistency values of α = 0.92 for the P-RCOPE subscale and α = 0.81 for the N-RCOPE subscale. In addition, the instrument has shown satisfactory concurrent, predictive, and incremental validity [15]. The Malay-translated version of the scale has also been validated, yielding Cronbach’s alpha coefficients of 0.87 for P-RCOPE and 0.88 for N-RCOPE [29]. Some sample items for P-RCOPE are: “Looked for a stronger connection with God” and “Focused on religion to stop worrying about my problems”. RCOPE is generally rated on a four-point Likert scale ranging from 0 (“not at all”) to 3 (“a great deal”), and the scores for positive and negative are summed up to give an overall score [15]. Participants completed the validated Malay version of Brief RCOPE. This translation includes culturally appropriate adaptations of references to “God” (e.g., “Tuhan/Allah” depending on context). The English sample items shown here are for illustration only and do not reflect the precise Malay wording used in the administered scale.

Furthermore, at the time of study design and data collection, a fully validated Malay version specifically adapted for Malaysian Muslim populations was not yet available. Therefore, the original Brief RCOPE was used, with minor linguistic adaptations to improve clarity and cultural relevance within the Malaysian context. These adaptations were conducted through expert review and pilot testing to ensure face validity and comprehensibility among participants. We acknowledge that a recently developed Malay version of the Brief RCOPE [22], which incorporates culturally and theologically nuanced adaptations for Muslim populations, may offer enhanced contextual validity. Future studies should consider employing this version to further strengthen cultural sensitivity and measurement precision.

### 2.9. Spirituality Assessment

Students’ spirituality was assessed using the Spirituality Scale (SS) developed by Jagers and Smith [30] (Supplementary File S4). The SS is a comprehensive instrument designed to evaluate multiple dimensions of spirituality, including beliefs, intuitions, lifestyle orientations, spiritual practices, and rituals that reflect the broader human spiritual experience. The scale was also developed to support the identification and guidance of spirituality-related interventions.

The instrument consists of 38 items rated on a six-point Likert scale ranging from 1 (“strongly disagree”) to 6 (“strongly agree”), with total scores calculated by summing responses across all items. The SS has demonstrated strong psychometric properties, with internal consistency coefficients ranging from α = 0.81 to 0.94 across its subscales and an overall reliability coefficient of α = 0.94 for the full instrument. In addition, the scale exhibited good test–retest reliability (*r* = 0.85) and satisfactory validity [31]. Examples of sample items include “I find meaning in my life experiences” and “My spirituality gives me inner strength”.

### 2.10. Spirituality Coping Assessment

Spiritual coping among students was evaluated using the Spiritual Coping Questionnaire (SCQ), a 32-item instrument comprising two principal dimensions: positive spiritual coping (P-SCQ) and negative spiritual coping (N-SCQ) [32] (Supplementary File S5). Responses are recorded using a five-point Likert scale. Both the P-SCQ and N-SCQ domains encompass four subdomains, namely personal, social, environmental, and religious coping. The questionnaire items assess different aspects of spiritual coping, allowing scores to be interpreted either as overall positive and negative coping tendencies or according to specific coping domains.

The SCQ has demonstrated satisfactory psychometric characteristics. The positive spiritual coping scale showed excellent internal consistency (α = 0.92), while the negative spiritual coping scale demonstrated good reliability (α = 0.82). Test–retest reliability coefficients were reported as *r* = 0.78 for the P-SCQ and *r* = 0.72 for the N-SCQ. Furthermore, the instrument exhibited good construct validity [32]. Examples of items are “I convinced myself that there is no purpose in my life”, “I convinced myself that my life has no meaning”, “I tried to look at the beauty and the uniqueness of nature”, and “In my relationship with God/higher power I looked for strength to live”.

### 2.11. Statistical Data Analysis

The collected data were analyzed using Statistical Package for Social Sciences (SPSS) software version 26 and SPSS Modeler version 18 (IBM Corp., Armonk, NY, USA). An alpha (α) was set at 0.05 with a confidence interval (CI) at 95%. Descriptive statistical analysis, such as mean, standard deviation, chi-square, and multiple linear regression, was applied to the data. The analysis was adjusted for covariates. The mediating variables investigated were religious and spiritual (positive and negative) coping i.e., the P-RCOPE, N-RCOPE, P-SCOPE, and N-SCOPE. Mediation analysis was conducted using a percentile bootstrap estimation approach with 10,000 resamples, implemented via the PROCESS Macro Version 4.1 (Model 4) [33], to examine whether religious and spiritual coping mediate the relationships between religiosity/spirituality and mental health outcomes (DASS-21: depression, anxiety, stress) as well as CCVD risk indicators. The use of bootstrapping enhances the robustness of estimates and statistical power, particularly in samples with moderate size and non-normal distributions.

To ensure coherence with the study’s pathway-based framework, statistical analyses were conducted in a structured, stepwise manner. Descriptive and chi-square analyses were first used to examine baseline associations between socio-demographic and clinical variables with mental health outcomes. Decision tree analysis (DTA) was subsequently applied as an exploratory, non-linear method to identify the relative importance and interaction of predictors. Several socio-demographic and clinical variables contained sparse subgroup counts (e.g., religion, ethnicity, marital status, chronic kidney disease, diabetes, and severe mental illness). Accordingly, chi-square and DTA findings involving these variables were interpreted cautiously and considered exploratory. Variables with extremely low frequencies were not interpreted as robust inferential predictors, and emphasis was placed on overall patterns and effect directions rather than isolated subgroup comparisons.

Multiple linear regression models were then used to quantify independent associations between religiosity, coping variables, and psychological outcomes. Finally, mediation analyses were conducted to test the central hypothesis that religious and spiritual coping mechanisms act as pathways linking religiosity/spirituality to mental health and CCVD risk indicators. This sequential approach was designed to integrate exploratory and hypothesis-driven analyses within a coherent analytical framework.

### 2.12. Ethical Considerations

The study protocol received ethical approval from the Universiti Sultan Zainal Abidin Human Research Ethics Committee (UHREC) under the reference code UniSZA/UHREC/2023/590. Written informed consent was obtained from all participants prior to study participation, and all collected data were anonymized to maintain participant confidentiality. Participants were also informed that their involvement was entirely voluntary and that they could withdraw from the study at any stage without any consequences or penalty.

## 3. Result

Results are presented in a structured sequence aligned with the study’s analytical framework, progressing from descriptive and exploratory analyses to hypothesis-driven regression and mediation models.

### 3.1. Clinical and Socio-Demographic Profiles

Table 1 summarizes the sociodemographic and clinical characteristics of 484 undergraduate students and their associations with depression, anxiety, and stress as measured by the DASS-21 scale. The mean age of participants was 21.26 years (SD = 3.07), and the majority were female (80.2%) and Malay (92.8%). Most students identified as Muslim (95.2%), were non-smokers (98.6%), and were single (97.3%). In terms of academic programs, the largest groups were from Medical and Health Sciences (21.7%) and Nursing (18.6%), with most participants in their first year of study (43.4%).

### 3.2. DASS-21 Profiles

Table 2 presents the distribution of depression, anxiety, and stress levels among the 484 students based on the DASS-21 scale. Regarding depression, 36.6% of students scored in the normal range, while 6.2% were classified as mild, 17.4% as moderate, 10.5% as severe, and 29.3% as extremely severe. For anxiety, only 21.5% were in the normal range, with 8.7% scoring mild, 9.5% moderate, 9.9% severe, and a striking 50.4% classified as extremely severe. In terms of stress, 30.8% of students fell within the normal range, followed by 11.0% mild, 18.4% moderate, 18.8% severe, and 21.1% extremely severe. These data indicate a notably high prevalence of moderate-to-extremely severe psychological distress among students, particularly for anxiety and stress.

### 3.3. Association Between DASS-21 and Clinical/Socio-Demographic Profiles

Based on Table 1, Chi-square analyses revealed significant associations between ethnicity and both anxiety (*p* < 0.001) and stress (*p* < 0.001), and between religion and anxiety (*p* < 0.001) as well as stress (*p* < 0.001). Associations involving ethnicity and religion should be interpreted as exploratory due to the highly imbalanced subgroup distribution within the sample. Program of study was significantly associated with depression (*p* < 0.001) and stress (*p* < 0.001). Although spousal status demonstrated a statistically significant association with anxiety (*p* < 0.05), this finding should be interpreted cautiously because only two participants reported being married, increasing the likelihood of unstable estimates and statistical inflation. However, given the limited representation within several subgroup categories, particularly for marital status, ethnicity, religion, and certain clinical conditions, the following analyses should be interpreted with caution, as sparse cell counts may reduce estimate stability and inflate apparent associations.

### 3.4. Associations Between DASS-21 Scores and CCVD-Related Risk Indicators

Among clinical variables, chronic kidney disease was significantly associated with anxiety (*p* = 0.0032), while a history of severe mental illness showed a significant association with stress (*p* = 0.006). Other clinical factors, including diabetes, hypertension treatment, migraines, and autoimmune conditions, did not show significant associations with the DASS-21 scales.

### 3.5. Predictor Importance and Decision Tree Analysis (DTA)

**Figure 1:**
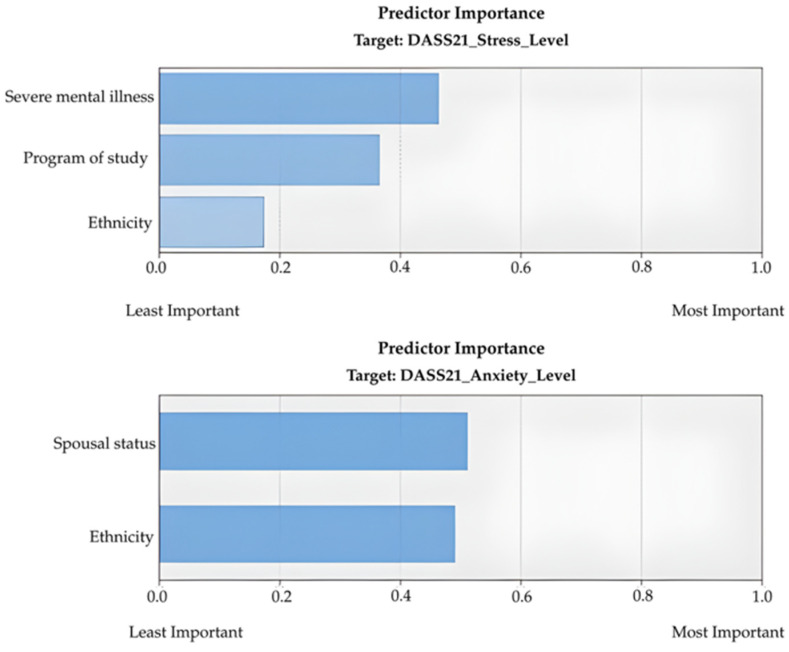
Predictor importance analysis for DASS-21 anxiety and stress outcomes based on decision tree analysis (DTA). The figure illustrates the relative contribution of socio-demographic and clinical variables to the exploratory prediction models. Depression was not included because no meaningful hierarchical splits were identified in the DTA model.

**Figure 2:**
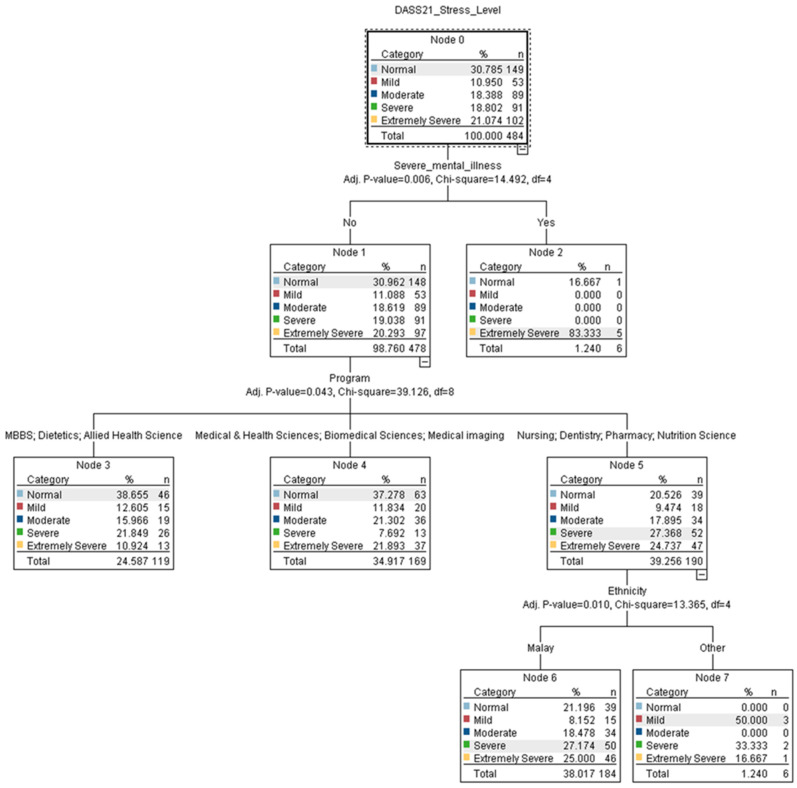
Decision tree analysis (DTA) illustrating the hierarchical predictor importance of the depression, anxiety, and stress 21 (DASS-21) stress level. The model identifies the relative importance and interaction of socio-demographic and clinical variables in predicting stress severity. Nodes represent decision splits, with the most influential predictor at the top. This exploratory model complements regression and mediation analyses by identifying non-linear relationships and subgroup patterns.

Based on the chi-square association analyses, independent variables including ethnicity, religion, program of study, spousal status, and the presence of chronic kidney disease or severe mental illness were identified for further evaluation as potential predictors of differences in DASS-21 scores among students. As illustrated in Figure 1, severe mental illness emerged as the most significant predictor of stress levels, followed by program of study and ethnicity. These associations were further supported by the detailed DTA results presented in Figure 2.

**Figure 3:**
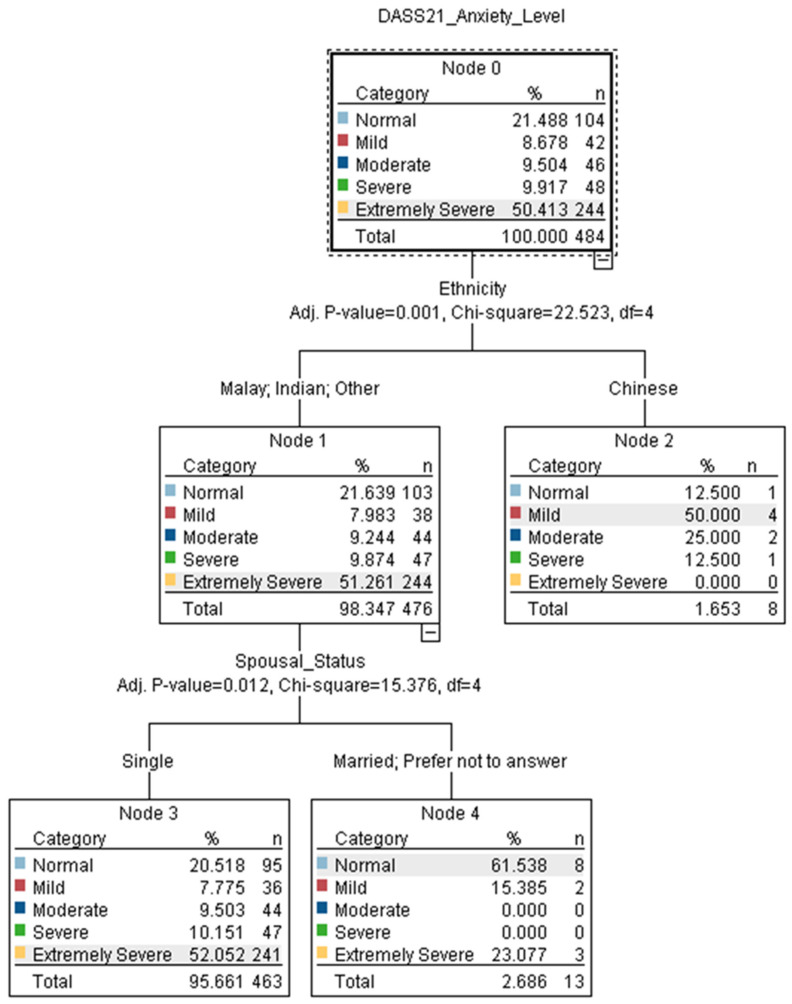
Decision tree analysis (DTA) model for the DASS-21 anxiety levels. The figure presents the exploratory hierarchical predictor structure associated with anxiety severity. Variables appearing higher in the tree indicate greater relative importance within the model. Findings are exploratory and not intended as confirmatory causal predictors.

Regarding anxiety levels, spousal status was identified as the most influential predictor, followed by ethnicity, with supporting evidence provided by the DTA output shown in Figure 3. For depression, both chi-square analysis and DTA identified program of study as the primary predictor. However, no additional meaningful splits were observed in the DTA model, indicating limited hierarchical or interaction effects among the examined variables. This suggests that unlike anxiety and stress, depression in this cohort was not strongly influenced by multiple interacting socio-demographic or clinical factors.

### 3.6. Association Between Religiosity and Religious Coping with DASS-21 and the Predictor Importance of CCVD-Related Risk Indicators

Table 3 presents the results of multiple linear regression analyses examining the relationship between the DASS-21 subscale scores (stress, anxiety, and depression) and three predictors: P-RCOPE, N-RCOPE, and religiosity (measured by the DUREL). For stress, both P-RCOPE and N-RCOPE were positively associated with stress levels, while religiosity was inversely related. A similar pattern was observed for anxiety, with significant positive associations for P-RCOPE and N-RCOPE, and a significant negative association for DUREL. In the model for depression, only N-RCOPE and DUREL were significantly associated, while P-RCOPE was not a significant predictor.

In the regression model examining the clinical indicators associated with CCVD vulnerability, such as severe mental illness, only N-RCOPE demonstrated a significant positive association. P-RCOPE and overall religiosity (DUREL) were not significantly associated with this risk factor. However, the model explained a very small proportion of the variance, indicating limited predictive value. With regard to ethnicity, exploratory analyses indicated a small association with positive religious coping (P-RCOPE), while negative religious coping (N-RCOPE) and overall religiosity (DUREL) were not significantly associated. However, this model explained only a minimal proportion of variance and should be interpreted with caution. Importantly, ethnicity in this study represents a background socio-cultural characteristic rather than an outcome variable. Given the highly skewed ethnic distribution of the sample (predominantly Malay), these findings are unlikely to reflect meaningful or generalizable relationships and are therefore considered exploratory.

The regression models for the DASS-21 subscales showed modest explanatory power, with adjusted R^2^ values ranging from 0.08 (Anxiety) to 0.156 (Depression), indicating that religiosity and religious coping styles accounted for only a small portion of the variance in psychological distress. Across all models, higher levels of religiosity were consistently associated with lower levels of stress, anxiety, and depression, suggesting a protective effect. In contrast, N-RCOPE was significantly associated with increased psychological distress, particularly depression. Interestingly, P-RCOPE, though typically considered adaptive, was positively associated with stress and anxiety, highlighting the possibility that such strategies may be insufficient or even counterproductive in certain contexts. These findings underscore the complex role of religious coping in mental health and the importance of distinguishing between its positive and negative dimensions in both research and clinical practice.

### 3.7. The Mediating Role of RCOPE in the Association Between Religiosity, DASS-21, and CCVD-Related Risk Indicators

Mediation analyses, which represent the primary hypothesis-testing component of this study, are presented below. Table 4 presents the mediation analysis assessing the role of religious coping (both positive and negative) in the relationship between religiosity and psychological distress (as measured by the DASS-21 subscales: stress, anxiety, and depression), as well as clinical indicators associated with CCVD vulnerability. The table displays the total, direct, and indirect effects, along with 95% CIs and corresponding t-statistics.

For stress, P-RCOPE significantly mediated the relationship between religiosity and stress levels, with a notable indirect effect, while the total effect was −0.55 (*p* < 0.001). In contrast, N-RCOPE did not mediate this relationship, as the indirect effect was negligible (−0.005) and the confidence interval crossed zero (−0.050 to 0.033). Similarly, for anxiety, P-RCOPE demonstrated a significant indirect effect, indicating partial mediation of the negative relationship between religiosity and anxiety. However, N-RCOPE again did not show significant mediation. For depression, the indirect effect of P-RCOPE was modest; however, the confidence interval crossed zero, indicating that the indirect association was not statistically significant. The total and direct effects remained strong and statistically significant. N-RCOPE had no mediating influence on the relationship between religiosity and depression.

Religiosity was positively associated with severe mental illness at the total effect level (β = 0.674, *p* < 0.001), but the direct effect became non-significant after controlling for positive religious coping, and the indirect effect through P-RCOPE was also non-significant. Similarly, the mediation path through negative religious coping showed no significant effects, with both direct and indirect effects being small and within a wide confidence interval that crossed zero. These results suggest that religious coping mechanisms do not meaningfully mediate the relationship between religiosity and severe mental illness as a CCVD risk factor in this sample. While the strong total effect might reflect complex psychological and behavioral dynamics in individuals with existing psychiatric diagnoses, these pathways appear to operate independently of spiritual coping styles.

Ethnicity, another predictor of clinical indicators associated with CCVD vulnerability, showed a small but significant total effect with religiosity (*p* < 0.001). However, the direct effect after accounting for P-RCOPE was attenuated, and the indirect effect through positive religious coping remained statistically significant. This indicates a modest mediation effect, where religious coping partially accounts for ethnic differences in reported religiosity levels. The mediation path through N-RCOPE was negligible and statistically non-significant. These findings may reflect underlying cultural or ethnoreligious variations in how religiosity is internalized and practiced. In particular, the role of positive religious coping as a partial mediator suggests that students from different ethnic backgrounds may engage with spirituality in distinct ways that influence their overall religiosity scores.

### 3.8. Association Between Spirituality and Spiritual Coping with DASS-21 and the Predictor Importance of CCVD-Related Risk Indicators

Table 5 summarizes the relationship between spirituality, spiritual coping, and mental health outcomes. Multiple regression analysis revealed that N-SCQ was a significant predictor of higher stress, anxiety, and depression scores on DASS-21. Notably, for stress, the association was negative; however, in anxiety and depression models, higher negative spiritual coping was consistently associated with greater psychological distress. P-SCQ was significantly associated with lower anxiety and depression scores, suggesting a modest protective effect. General spirituality was not a significant predictor in any of the mental health models. The models accounted for limited variance (R^2^ range: 0.027–0.084), with the highest explanatory power observed in the depression model (adjusted R^2^ = 0.078). In contrast, spirituality, positive, and negative spiritual coping did not significantly predict the CCVD risk factor, i.e., severe mental illness (R^2^ = 0.007; adjusted R^2^ = 0.001) or ethnicity (R^2^ = 0.006; adjusted R^2^ = 0.000), and the regression equations were non-informative.

### 3.9. The Mediating Role of SCQ in the Association Between Spirituality, DASS-21, and CCVD-Related Risk Indicators

Table 6 summarizes the mediation analysis examining the role of spiritual coping, both positive (P-SCQ) and negative (N-SCQ), in the relationship between spirituality (SS), psychological distress (as measured by DASS-21 subscales), and CCVD-related risk indicators. The table presents the total, direct, and indirect effects, 95% CIs, and t-statistics for each pathway.

For DASS-21 (stress), N-SCQ’s 95% CI (0.001 to 0.047) was entirely above zero, indicating a significant mediating effect. However, the indirect effect of P-SCQ had a CI (−0.003 to 0.070) that crossed zero, indicating a non-significant indirect association. For DASS-21 (anxiety), both coping styles again demonstrated modest indirect effects (P-SCQ: 0.02; N-SCQ: 0.01), with N-SCQ showing a statistically significant mediating role (95% CI: 0.001 to 0.042), while the CI for P-SCQ included zero (−0.004 to 0.069), indicating a non-significant indirect effect. In the case of DASS-21 (depression), both P-SCQ and N-SCQ significantly mediated the relationship between spirituality and depressive symptoms. The indirect effects were 0.03 for both, with CIs that did not include zero (P-SCQ: 0.001 to 0.102; N-SCQ: 0.001 to 0.068), suggesting that both positive and negative spiritual coping mechanisms play meaningful roles in this association.

For predictor importance of clinical indicators associated with CCVD vulnerability, such as severe mental illness, the total effect of spirituality was significant. However, the direct effects through both P-SCQ and N-SCQ were not statistically significant (*p* = 0.26 and *p* = 0.18, respectively), indicating a full mediation effect via coping pathways. The indirect effects through both P-SCQ and N-SCQ were modest, with confidence intervals crossing zero, suggesting a weak but possible mediating role of spiritual coping in linking spirituality to cardiovascular risk via mental illness. The model shows that spirituality may influence mental illness scores indirectly through coping mechanisms, despite nonsignificant direct paths.

In contrast, for ethnicity, spirituality had a significant total effect (*p* < 0.001), but both direct and indirect effects were non-significant (P-SCQ direct effect: *p* = 0.56; indirect effect: ~0; N-SCQ direct effect: *p* = 0.54; indirect effect: ~0), with confidence intervals including zero. The analysis involving ethnicity should be interpreted with caution. Given the highly imbalanced ethnic composition of the sample, the observed associations are unlikely to represent meaningful or generalizable relationships. Rather than indicating a predictive role of religiosity or coping, these findings likely reflect underlying socio-cultural patterns or sampling effects. Ethnicity is more appropriately conceptualized as a contextual variable shaping religious expression and coping styles, rather than as an outcome influenced by these factors.

## 4. Discussion

This study was designed to test a pathway-based biopsychosocial framework in which religiosity and spirituality influence mental health through coping mechanisms, rather than acting as direct protective factors, and may thereby contribute to early vulnerability to CCVD. The use of multiple analytical approaches allowed for both exploratory pattern detection and hypothesis testing; however, these methods were applied within a structured framework to examine complementary aspects of the same underlying pathway. Our findings provide partial support for this framework, demonstrating that the effects of religiosity and spirituality are contingent on coping style, with adaptive strategies associated with better psychological outcomes and maladaptive strategies linked to greater distress. These results highlight the complex and potentially bidirectional roles of coping in shaping both mental health and early indicators of physical health risk.

### 4.1. Mental Health Burden

This study provides a comprehensive investigation into the interrelationships between religiosity, spirituality, religious/spiritual coping, psychological distress, and early CCVD risk factors among a young cohort of multiethnic Southeast Asian, i.e., Malaysian, undergraduate students in health-related programs. Our findings not only reveal a high prevalence of psychological distress, especially anxiety and stress, but also highlight the nuanced and differential roles regarding the role of religious and spiritual coping strategies in influencing mental health outcomes. These findings are especially pertinent within the Malaysian sociocultural context, where religion and spirituality are deeply embedded in cultural identity.

The alarmingly high prevalence of moderate to extremely severe anxiety (69.7%) and stress (59.3%) in our sample underscores a growing mental health burden among university students. This is consistent with recent global data showing elevated psychological morbidity in post-pandemic academic environments, particularly among healthcare trainees who face unique academic, emotional, and clinical stressors [36,37]. The disproportionate burden among females and early-year students further supports previous literature indicating that younger students may lack coping maturity or adequate support structures [38].

Our findings identified important socio-clinical predictors, notably ethnicity, religion, program of study, spousal status, and history of severe mental illness, mirroring multifactorial determinants of distress. These findings suggest that interventions targeting mental health in university settings must be demographically and culturally contextualized, particularly in multi-ethnic nations like Malaysia. Moreover, the association between chronic kidney disease and anxiety, and between severe mental illness and stress, while not novel, reinforces the bidirectional nature of physical and mental health comorbidities [39]. However, these results should be interpreted cautiously and considered exploratory.

### 4.2. Religiosity, Spirituality, and Coping Mechanisms

The central innovation of this study is the dual-pathway mediation model exploring religious and spiritual coping as intermediaries between religiosity/spirituality and mental health outcomes. Among the analytical approaches employed, mediation analysis provides the most direct support for the study’s central hypothesis, demonstrating that coping mechanisms function as key pathways linking religiosity and spirituality to psychological outcomes. While higher religiosity and spirituality were consistently associated with lower levels of depression, anxiety, and stress, the mechanisms underlying this relationship were heterogeneous and nuanced [13,40]. Our findings show that positive religious coping significantly mediated the inverse association between religiosity and both stress and anxiety, suggesting that religious practices such as prayer, spiritual surrender, and reframing suffering through a sacred lens may facilitate emotion-focused coping and resilience [15,18,41]. However, surprisingly, positive religious coping was also positively associated with higher distress in regression models, highlighting a paradoxical effect. This may reflect a maladaptive reliance on religious coping in lieu of active problem-solving, a phenomenon reported in other collectivist religious societies where passive religious coping may delay help-seeking [42].

Conversely, negative religious coping, characterized by spiritual discontent, punishment-based beliefs, and interpersonal religious strain, was consistently associated with increased psychological distress and did not mediate any beneficial effects [43,44]. These findings are consistent with meta-analytic evidence linking negative religious coping to adverse outcomes across diverse populations [45], underscoring the need to screen for dysfunctional religious coping in mental health settings. Equally noteworthy is our examination of spiritual coping, a construct less frequently studied in Asian contexts. Both positive and negative spiritual coping significantly mediated the relationship between spirituality and depression, with negative spiritual coping also emerging as a significant mediator for stress and anxiety. This suggests that spirituality, distinct from organized religiosity, operates through both constructive and maladaptive mechanisms that influence mental health trajectories. Therefore, the dual mediation roles of both positive and negative spiritual coping highlight a novel insight: even individuals with strong spiritual orientations may experience distress if their coping mechanisms involve existential struggle, guilt, or disconnection from transcendent beliefs. This is congruent with growing literature on spiritual struggles and their association with depressive symptoms, even among the highly spiritual [46,47].

An important consideration arising from our findings is the distinction between religiosity, spirituality, and their respective coping mechanisms. While these constructs are often used interchangeably, they represent overlapping yet non-identical domains. Religiosity refers primarily to participation in and commitment to organized beliefs and practices, whereas spirituality reflects a broader, more individualized search for meaning, connection, and transcendence [11,12]. Similarly, religious coping emphasizes the use of faith-based strategies such as prayer or reliance on divine support, whereas spiritual coping mechanisms encompass existential and meaning-oriented approaches that may or may not be tied to institutionalized religion [15,16]. Recognizing these distinctions is crucial, particularly in multicultural contexts such as Malaysia, where multiple religious traditions coexist, and spirituality may extend beyond formal doctrine. Furthermore, cultural nuances in religious interpretation and expression, particularly in Muslim-majority settings, may influence how coping strategies are conceptualized and reported. This further underscores the importance of culturally adapted instruments such as the Brief RCOPE-Malay in future investigations. Finally, our results highlight that while religiosity and spirituality are generally associated with protective patterns, their coping expressions (positive vs. negative) can diverge, at times producing paradoxical or even maladaptive outcomes. Clarifying these conceptual boundaries not only enhances the interpretability of our findings but also underscores the need for culturally sensitive frameworks that differentiate between institutional and existential pathways to resilience.

### 4.3. Links to CCVD Risk

Beyond mental health, we also explored early indicators of physical health vulnerability, specifically the predictor importance of vascular risk factors. While overall vascular burden was low in this young sample, severe mental illness and ethnic background were associated with early CCVD risk profiles in exploratory analyses. Notably, participants with a known history of psychiatric illness showed greater psychological distress and also contributed disproportionately to vascular risk clusters, consistent with existing literature linking chronic mental illness to long-term cardiovascular morbidity via inflammatory and behavioral pathways [48,49]. Although religiosity and spirituality have been implicated in cardiometabolic outcomes among older adults [50,51,52], the lack of association in our sample suggests that these constructs may have limited predictive value for CCVD in medically literate, low-risk youth cohorts.

Additionally, ethnic disparities in CCVD risk factor distribution were observed, which echo broader public health patterns in Malaysia and Southeast Asia. These findings suggest that ethnicity likely acts as a proxy for complex sociocultural and structural variables that may influence both the perception of distress and access to preventive healthcare [53]. More nuanced exploration of these intersectional dynamics is warranted in future work. However, the apparent association with ethnicity should be interpreted cautiously, as the lack of ethnic diversity in the sample limits statistical power and may produce unstable or non-generalizable estimates. Importantly, the sex distribution of our sample, which was predominantly female (~80%), may have influenced the observed patterns of religious coping and mental health outcomes. Previous studies have consistently shown that female students report higher rates of anxiety and depression, as well as greater reliance on religious and spiritual coping mechanisms compared to their male counterparts [38,54,55,56,57]. Women are also more likely to seek emotional expression through faith, prayer, and meaning-making during stress, which may enhance the salience of positive spiritual coping strategies in this sample [58,59]. Conversely, heightened emotional sensitivity may also make female students more susceptible to maladaptive forms of religious struggle, such as guilt or divine abandonment, which aligns with our findings on negative coping [60]. These gendered patterns in coping and distress should be further investigated in more balanced or stratified samples.

### 4.4. Strength and Implication

Finally, the high internal reliability of all adapted instruments confirms the applicability of spiritual and religious measures in this population. By establishing these tools’ utility in a multiethnic Malaysian context, this study contributes methodologically to the global health psychology and psychiatry literature. In summary, our findings suggest that adaptive spiritual frameworks and coping styles can play a protective role in mental well-being and may help lower the early-life CCVD risk, especially among vulnerable subgroups. Integrating culturally informed, spiritually sensitive approaches into student mental health services may enhance psychological resilience and support holistic well-being in diverse academic settings.

Moreover, these findings have practical and policy implications. First, screening tools for spiritual and religious coping styles could be integrated into student wellness programs to identify individuals at risk of maladaptive coping. Second, training for campus mental health providers should include culturally sensitive approaches to assess and manage religious or spiritual concerns. Third, our mediation framework provides a model for culturally embedded mental health interventions that incorporate faith-based resources, particularly in Muslim-majority countries.

### 4.5. Study Limitations and Future Approaches

This study has several limitations. First, the cross-sectional nature of the study precludes the establishment of causal relationships; therefore, longitudinal investigations are warranted to better elucidate the temporal associations among the variables examined. Furthermore, the achieved sample size was smaller than the initially calculated target, which may have reduced statistical power to detect small effect sizes. While the overall sample remained sufficient for primary analyses involving continuous variables, subgroup analyses (e.g., ethnicity, clinical conditions) involved small category sizes, increasing the risk of unstable estimates and limiting generalizability. Therefore, findings related to these subgroups should be interpreted as exploratory.

Second, the reliance on self-report questionnaires may introduce response biases. Third, while the sample was diverse in terms of study program and religion, it was predominantly female and Malay, limiting generalizability. Additionally, the analysis involving ethnicity is limited by the highly skewed sample composition, with a predominance of Malay participants (92.8%). This imbalance restricts the interpretability of ethnicity-related findings and increases the risk of spurious or non-generalizable associations. Therefore, these results should be interpreted cautiously and considered exploratory. Future studies with more ethnically balanced samples are needed to meaningfully explore the role of cultural diversity in shaping religiosity, spirituality, and coping mechanisms. Fourth, while our study focused on self-reported and clinical indicators of early vascular risk (e.g., chronic illness, family history), future research should consider incorporating objective physiological markers of physical health and stress. For instance, blood pressure, heart rate variability, body composition, salivary cortisol, and C-reactive protein (CRP) could provide a more accurate picture of psychophysiological functioning. These biomarkers would allow researchers to capture the biological correlates of chronic stress and explore whether spiritual coping influences not only subjective distress but also stress-related physiological dysregulation such as allostatic load. Integrating these measures could significantly enhance the translational value of future studies.

The study did not employ validated CCVD prediction algorithms or objective physiological cardiovascular measurements. Instead, CCVD-related outcomes were based on self-reported clinical risk indicators, which limits causal inference and may overestimate the strength of cardiovascular implications. Furthermore, although spousal status emerged as the most influential predictor of anxiety in the DTA, this finding must be interpreted with caution. The vast majority of the sample were single (97.3%), with only two participants reporting that they were married. This substantial demographic imbalance increases the risk of statistical inflation, whereby very small subgroups can appear disproportionately influential in machine-learning-based models such as DTA. Therefore, the observed association between marital status and anxiety is unlikely to reflect a true population-level pattern and should not be over-interpreted. Future studies with more balanced representation across marital status categories are needed to determine whether spouse-related factors genuinely influence anxiety risk in student populations.

Another limitation relates to the measurement of religious coping. Although Brief RCOPE is a globally validated instrument, the version used in this study involved a minor linguistic adaptation rather than a formally validated Malay version. A recently developed Brief RCOPE-Malay [22], specifically tailored to Muslim populations, may provide improved cultural and theological sensitivity. The absence of this instrument at the time of data collection may limit the contextual precision of our findings. Future studies should incorporate such culturally validated tools to enhance measurement accuracy and cross-cultural applicability.

Future research should pursue longitudinal designs to examine how coping styles evolve over time and their impact on mental and physical health trajectories. Mixed-methods studies may provide deeper insights into the lived experience of religious and spiritual coping. Incorporating biomarkers and digital phenotyping could bridge the gap between subjective distress and objective health risks. Additionally, intervention studies assessing the impact of spiritual care, chaplaincy support, or faith-integrated cognitive behavior therapy (CBT) could offer evidence-based strategies for clinical application.

## 5. Conclusions

This study provides evidence for a biopsychosocial pathway linking religiosity, spirituality, coping mechanisms, and mental health with early indicators of CCVD risk. Our findings demonstrate that while religiosity and spirituality are generally associated with lower psychological distress, their effects are contingent on coping style: positive coping appears to promote psychological resilience, whereas negative coping is consistently associated with poorer mental health outcomes. Mediation analyses further suggest that coping mechanisms act as key pathways through which existential beliefs influence mental well-being. Importantly, psychological distress, particularly among individuals with severe mental illness, was associated with a greater burden of self-reported CCVD-related risk indicators, supporting the role of mental health as an upstream determinant of cardiovascular vulnerability. Observed sex-related patterns in coping and distress further highlight the need for culturally and demographically sensitive approaches. Overall, these findings underscore the potential of spiritually informed, evidence-based strategies as part of integrated mental health and early prevention frameworks for non-communicable diseases. Targeting maladaptive coping while strengthening adaptive spiritual resources may represent a practical and culturally relevant pathway to enhance resilience and reduce long-term cardiovascular risk in young adult populations.

## Figures and Tables

**Table 1:** Students’ sociodemographic profiles and clinical proforma (*N* = 484) and association with the DASS-21 scales.

Socio-Demography Variables	Frequency, *N* (%)	Chi-Square; *X*^2^ (df)		
		Depression	Anxiety	Stress
Age **	21.26 ± 3.07	53.49 (40)	41.38 (40)	40.33 (40)
Gender				
Male	86 (17.8)			
Female	388 (80.2)	4.08 (8)	8.52 (8)	10.88 (8)
Undisclosed	10 (2.1)			
Ethnicity				
Malay	449 (92.8)			
Chinese	8 (1.7)	10.67 (12)	27.89 (12) *	21.28 (12) *
Indian	12 (2.5)			
Other	15 (3.1)			
Religion				
Islam	461 (95.2)			
Buddhism	4 (0.8)	21.37 (20)	45.18 (20) *	32.34 (20) *
Taoism	1 (0.2)			
Hinduism	13 (2.7)			
Sikhism	3 (0.6)			
Others	2 (0.4)			
Smoking Status				
Non-smoker	477 (98.6)	4.69 (8)	13.35 (8)	14.14 (8)
Ex-smoker	6 (1.2)			
Light smoker	1 (0.2)			
Spousal Status				
Single	471 (97.3)	6.87 (8)	17.76 (8) *	7.83 (8)
Married	2 (0.4)			
Undisclosed	11 (2.3)			
Program				
MBBS	48 (9.9)			
Medical & Health Sc.	105 (21.7)			
Nursing	90 (18.6)			
Dentistry	5 (1.0)	57.84 (36) *	46.18 (36)	66.66 (36) *
Pharmacy	53 (11)			
Dietetics	50 (10.3)			
Biomedical sciences	29 (6)			
Nutrition sciences	46 (9.5)			
Medical imaging	36 (7.4)			
Allied Health Sciences	22 (4.5)			
Level of Study				
Year 1	210 (43.4)			
Year 2	130 (26.9)	21.09 (160)	24.79 (16)	26.11 (16)
Year 3	111 (22.9)			
Year 4	32 (6.6)			
Year 5	1 (0.2)			
**Clinical Proforma**				
Diabetes status				
None	478 (98.8)			
Type 1	3 (0.6)	4.38 (8)	15.00 (8)	9.76 (8)
Type 2	3 (0.6)			
Family history of heart disease				
No	475 (98.1)	3.44 (4)	2.88 (4)	1.94 (4)
Yes	9 (1.9)			
Chronic kidney disease				
No	483 (99.8)	8.51 (4)	10.55 (4) *	4.45 (4)
Yes	1 (0.2)			
Atrial fibrillation				
No	484 (100)	N/A	N/A	N/A
Yes	0 (0)			
On blood pressure treatment				
No	481 (99.4)	2.58 (4)	6.76 (4)	3.16 (4)
Yes	3 (0.6)			
Migraines				
No	408 (84.3)	8.37 (4)	5.86 (4)	1.08 (4)
Yes	76 (15.7)			
Rheumatoid arthritis				
No	482 (99.6)	3.48 (4)	7.34 (4)	4.52 (4)
Yes	2 (0.4)			
Systemic lupus erythematosus				
No	484 (100)	N/A	N/A	N/A
Yes	0 (0)			
Severe mental illness				
No	478 (98.8)	5.19 (4)	3.08 (4)	14.50 (4) *
Yes	6 (1.2)			
On antipsychotic medication				
No	482 (99.6)	4.13 (4)	1.32 (4)	2.00 (4)
Yes	2 (0.4)			
On steroid tablets				
No	483 (99.8)	1.74 (4)	3.66 (4)	2.25 (4)
Yes	1 (0.2)			
Erectile dysfunction				
No	484 (100)	N/A	N/A	N/A
Yes	0 (0)			

Notes: data values are presented as number of subjects (n), with percentage (%) in parentheses; * significant at *p* < 0.05; ** Data are means ± standard deviations (SD). Light smoker (≤10 per day); severe mental illness includes schizophrenia, bipolar disorder, and 2/3 depression. Data presented in this table were adapted and refined from Sukeri et al. [34] and Mazzuki et al. [35].

**Table 2:** Students’ Depression, Anxiety, and Stress 21 (DASS-21) profile.

DASS-21 Scoring	Frequency, *N* (%)		
	Depression	Anxiety	Stress
Normal	177 (36.6)	104 (21.5)	149 (30.8)
Mild	30 (6.2)	42 (8.7)	53 (11)
Moderate	84 (17.4)	46 (9.5)	89 (18.4)
Severe	51 (10.5)	48 (9.9)	91 (18.8)
Extremely Severe	142 (29.3)	244 (50.4)	102 (21.1)

Data presented in this table were adapted and refined from Sukeri et al. [34] and Mazzuki et al. [35].

**Table 3:** Multiple linear regression models examining the associations between religiosity (DUREL), religious coping (P-RCOPE, N-RCOPE), and psychological distress (DASS-21 subscales: stress, anxiety, depression) as well as selected CCVD-related risk indicators.

Dependent Variables		Independent Variables	Std. Coefficient Beta (β)
**DASS-21 Stress (*y*)**		Constant	2.692
		P-RCOPE *x*_1_	0.203 *
		N-RCOPE *x*_2_	0.169 *
		DUREL *x*_3_	−0.368 *
*R* ^2^	0.116		
Adjusted *R*^2^	0.111		
Regression equation *ŷ* = 2.692 + 0.203 *x*_1_ + 0.169 *x*_2_ − 0.368 *x*_3_			
**DASS-21 Anxiety (*y*)**		Constant	2.769
		P-RCOPE *x*_1_	0.157 *
		N-RCOPE *x*_2_	0.181 *
		DUREL *x*_3_	−0.277 *
*R* ^2^	0.08		
Adjusted *R*^2^	0.08		
Regression equation *ŷ* = 2.769 + 0.157 *x*_1_ + 0.181 *x*_2_ − 0.277 *x*_3_			
**DASS-21 Depression (*y*)**		Constant	2.820
		P-RCOPE *x*_1_	0.075
		N-RCOPE *x*_2_	0.244 *
		DUREL *x*_3_	−0.354 *
*R* ^2^	0.162		
Adjusted *R*^2^	0.156		
Regression equation *ŷ* = 2.820 + 0.244 *x*_2_ − 0.354 *x*_3_			
**Severe Mental Illness (*y*)**		Constant	0.007
		P-RCOPE *x*_1_	−0.001
		N-RCOPE *x*_2_	0.005 *
		DUREL *x*_3_	0.002
*R* ^2^	0.017		
Adjusted *R*^2^	0.011		
Regression equation *ŷ* = 0.007 + 0.005 *x*_2_			
**Ethnicity (*y*)**		Constant	0.534
		P-RCOPE *x*_1_	−0.030 *
		N-RCOPE *x*_2_	*p* < 0.001
		DUREL *x*_3_	0.017
*R* ^2^	0.018		
Adjusted *R*^2^	0.012		
Regression equation *ŷ* = 0.534 − 0.030 *x*_1_			

Standardized beta coefficients (β) are reported. Positive β values indicate higher levels of distress with increasing predictor values, while negative β values indicate protective associations. Models are adjusted for relevant covariates. * *p* < 0.05 indicates statistical significance.

**Table 4:** Summary of religious coping mediation in religiosity, DASS-21, and CCVD-related risk indicators.

Relationship	Total Effect	Direct Effect	Indirect Effect	CI at 95%		T-Statistic
				Lower	Upper	
**DASS-21 Stress**						
Religiosity → P-RCOPE → Stress	−0.55 (0.00)	−0.88 (0.00)	0.336	0.161	0.510	−5.43
Religiosity → N-RCOPE → Stress	−0.55 (0.00)	−0.55 (0.00)	−0.005	−0.050	0.033	−5.47
**DASS-21 Anxiety**						
Religiosity → P-RCOPE → Anxiety	−0.41 (0.00)	−0.68 (0.00)	0.275	0.100	0.460	−3.98
Religiosity → N-RCOPE → Anxiety	−0.41 (0.00)	−0.41 (0.00)	−0.005	−0.052	0.034	−3.98
**DASS-21 Depression**						
Religiosity → P-RCOPE → Depression	−0.76 (0.00)	−0.94 (0.00)	0.177	−0.019	0.363	−7.14
Religiosity → N-RCOPE → Depression	−0.76 (0.00)	−0.75 (0.00)	−0.007	−0.068	0.047	−7.14
**Severe Mental Illness (SMI)**						
Religiosity → P-RCOPE → SMI	0.674 (0.00)	−0.018 (0.905)	0.129	0.141	0.297	−18.039
Religiosity → N-RCOPE → SMI	0.011 (0.793)	0.092 (0.428)	−0.004	−0.049	0.038	−0.262
**Ethnicity**						
Religiosity → P-RCOPE → Ethnicity	0.067 (0.00)	−0.017 (0.107)	−0.020	−0.035	−0.006	1.615
Religiosity → N-RCOPE → Ethnicity	−0.011 (0.793)	−0.003 (0.708)	0.000	−0.001	−0.001	−0.0262

CCVD, cardio-cerebrovascular risk; CI, confidence interval; DASS-21, depression-anxiety-stress scale-21; → indicate “mediating role”.

**Table 5:** Multiple linear regression of the relationship between spirituality and spiritual coping with DASS-21 and predictor importance for cardio-cerebrovascular (CCVD)-related risk indicators.

Dependent Variables		Independent Variables	Std. Coefficient Beta (β)
**DASS-21 Stress (*y*)**		Constant	10.079
		P-SCQ *x*_1_	−0.133
		N-SCQ *x*_2_	−0.590 *
		SS *x*_3_	−0.133
*R* ^2^	0.042		
Adjusted *R*^2^	0.036		
Regression equation *ŷ* = 10.079 − 0.590 *x*_2_			
**DASS-21 Anxiety (*y*)**		Constant	7.854
		P-SCQ *x*_1_	−0.131 *
		N-SCQ *x*_2_	0.455 *
		SS *x*_3_	0.021
*R* ^2^	0.027		
Adjusted *R*^2^	0.021		
Regression equation *ŷ* = 7.854 − 0.131 *x*_1_ + 0.455 *x*_2_			
**DASS-21 Depression (*y*)**		Constant	6.270
		P-SCQ *x*_1_	−0.146 *
		N-SCQ *x*_2_	0.882 *
		SS *x*_3_	−0.231
*R* ^2^	0.084		
Adjusted *R*^2^	0.078		
Regression equation *ŷ* = 6.270 − 0.146 *x*_1_ + 0.882 *x*_2_			
**Severe Mental Illness (*y*)**		Constant	−0.053
		P-SCQ *x*_1_	−0.000
		N-SCQ *x*_2_	0.001
		SS *x*_3_	0.001
*R* ^2^	0.007		
Adjusted *R*^2^	0.001		
Regression equation *ŷ* = Regression equation not reported due to non-significant model.			
**Ethnicity (*y*)**		Constant	0.300
		P-SCQ *x*_1_	0.004
		N-SCQ *x*_2_	−0.016
		SS *x*_3_	−0.001
*R* ^2^	0.006		
Adjusted *R*^2^	0.000		
Regression equation *ŷ* = Regression equation not reported due to non-significant model.			

Standardized beta coefficients (β) are reported. Positive β values indicate higher levels of distress with increasing predictor values, while negative β values indicate protective associations. Models are adjusted for relevant covariates. * *p* < 0.05 indicates statistical significance.

**Table 6:** Summary of spiritual coping mediation in spirituality (SS), DASS-21, and predictor importance of CCVD-related risk indicators.

Relationship	Total Effect	Direct Effect	Indirect Effect	CI at 95%		T-Statistic
				Lower	Upper	
**DASS-21 Stress**						
SS → P-SCQ → Stress	−0.22 (0.00)	−0.23 (0.00)	0.02	−0.003	0.070	−5.35
SS → N-SCQ → Stress	−0.21 (0.00)	−0.23 (0.00)	0.02	0.001	0.047	−5.35
**DASS-21 Anxiety**						
SS → P-SCQ → Anxiety	−0.17 (0.00)	−0.19 (0.00)	0.02	−0.004	0.069	−4.204
SS → N-SCQ → Anxiety	−0.17 (0.00)	−0.18 (0.00)	0.01	0.001	0.042	−4.204
**DASS-21 Depression**						
SS → P-SCQ → Depression	−0.28 (0.00)	−0.31 (0.00)	0.03	0.001	0.102	−6.632
SS → N-SCQ → Depression	−0.28 (0.00)	−0.31 (0.00)	0.03	0.001	0.068	−6.632
**Severe mental illness**						
SS → P-SCQ → Severe mental illness	0.29 (0.00)	−0.034 (0.26)	0.013	−0.017	0.061	5.432
SS → N-SCQ → severe mental illness	0.06 (0.01)	−0.041 (0.18)	0.009	−0.005	0.032	2.548
**Ethnicity**						
SS → P-SCQ → ethnicity	0.29 (0.00)	0.002 (0.56)	−0.000	−0.003	0.001	5.432
SS → N-SCQ → ethnicity	0.059 (0.01)	0.002 (0.54)	−0.000	−0.002	0.000	2.548

CCVD, cardio-cerebrovascular risk; CI, confidence interval; DASS-21, depression-anxiety-stress scale-21; → indicate “mediating role”.

## Data Availability

The raw data supporting the conclusions of this article will be made available by the authors on request.

## Supplementary Materials

The following supporting information can be downloaded at: https://www.mdpi.com/article/10.3390/ijerph23060738/s1, File S1: DASS-21 [61]; File S2: The Duke University Religion Index (DUREL); File S3: The Brief RCOPE; File S4: The Spirituality Scale; File S5: The Spiritual Coping Questionnaire (SCQ).

## Abbreviations

The following abbreviations are used in this manuscript:

CBT	Cognitive behavior therapy
CCVD	Cardio-cerebrovascular disease
CI	Confidence interval
CRP	C-reactive protein
DASS-21	Depression, Anxiety, and Stress Scale
DTA	Decision tree analysis
DUREL	Duke university religion index
HPA	Hypothalamic–pituitary–adrenal
IR	Intrinsic religiosity
NORA	Non-organized religious activity
N-RCOPE	Negative religious coping
N-SCQ	Negative spiritual coping
ORA	Organized religious activity
P-RCOPE	Positive religious coping
P-SCQ	Positive spiritual coping
RCOPE	Brief religious coping
SCQ	Spiritual coping questionnaire
SD	Standard deviation
SS	Spirituality scale
