# Supplementary material for: Coping as a Pathway Linking Religiosity and Spirituality to Mental Health and Early Cardio-Cerebrovascular Risk Among University Students in Malaysia
Source: Int J Environ Res Public Health. 2026 May 31;23(6):738. doi: 10.3390/ijerph23060738 (PMC13299289; doi:10.3390/ijerph23060738)
Supplement: Supplementary file 1 [file ijerph-23-00738-s001.zip › Supplementary File S2.pdf]

Supplementary File S2

The Duke University Religion Index (DUREL)

1 = Never; 2 = Once a year or less; 3 = A few times a year; 4 = A few times a month; 5 = Once a week; 6 = More than once a week

	1	2	3	4	5	6
1. How often do you attend, mosque, surau, temple, church, or other religious meetings?						
2. How often do you spend time in private religious activities, such as prayer, meditation, religious book reading and the study of religious books?						

The following 3 statements are rated from 1 - 5. 1. Definitely not true, 2. Tends not to be true, 3. Unsure, 4. Tends to be true, 5. Definitely true of me.

	1	2	3	4	5
3. In my life I feel or experience the presence of the Devine (i.e. Allah, God, etc.)					
4. My religious beliefs are what really lie behind my whole approach to life					
5. I try hard to carry my religion over into all other dealings in life.					
