# Supplementary material for: Coping as a Pathway Linking Religiosity and Spirituality to Mental Health and Early Cardio-Cerebrovascular Risk Among University Students in Malaysia
Source: Int J Environ Res Public Health. 2026 May 31;23(6):738. doi: 10.3390/ijerph23060738 (PMC13299289; doi:10.3390/ijerph23060738)
Supplement: Supplementary file 1 [file ijerph-23-00738-s001.zip › Supplementary File S3.pdf]

Supplementary File S3

The Brief RCOPE

1 – Not at all
2 – Somewhat
3 – Quite a bit
4 – A great deal

	1	2	3	4
1. I look for a stronger connection with the God.				
2. I sought the God's love and care.				
3. I sought help from the God in letting go of my anger.				
4. I tried to put my plans into action together with the God.				
5. I tried to see how the God might be trying to strengthen me in this situation.				
6. I asked forgiveness for my sins.				
7. I focused on religion to stop worrying about my problems.				
8. I wondered whether the God had abandoned me.				
9. I felt punished by the God for my lack of devotion.				
10. I wondered what I did for the God to punish me.				
11. I questioned the God's love for me.				
12. I wondered whether my religious group had abandoned me.				
13. I decided the devil made this happen.				
14. I questioned the power of the God.				
