# Supplementary material for: Coping as a Pathway Linking Religiosity and Spirituality to Mental Health and Early Cardio-Cerebrovascular Risk Among University Students in Malaysia
Source: Int J Environ Res Public Health. 2026 May 31;23(6):738. doi: 10.3390/ijerph23060738 (PMC13299289; doi:10.3390/ijerph23060738)
Supplement: Supplementary file 1 [file ijerph-23-00738-s001.zip › Supplementary File S4.pdf]

Supplementary File S4

The Spirituality Scale

1. Strongly Disagree
2. Disagree
3. Mostly disagree
4. Mostly agree
5. Agree
6. Strongly Agree

	1	2	3	4	5	6
1. I find meaning in my life experiences.						
2. I have a sense of purpose.						
3. I am happy about the person I have become.						
4. I see the sacredness in everyday life.						
5. I meditate/do zikir/contemplate to gain access to my inner spirit						
6. I live in harmony with nature.						
7. I believe there is a connection between all things that I cannot see but can sense.						
8. My life is a process of becoming.						
9. I believe in a Higher Power/Universal Intelligence.						
10. I believe that all living creatures deserve respect.						
11. The earth is sacred.						
12. I value maintaining and nurturing my relationships with others.						
13. I use silence to get in touch with myself.						
14. I believe that nature should be respected.						
15. I have a relationship with a Higher Power/Universal Intelligence.						
16. My spirituality gives me inner strength.						
17. I am able to receive love from others.						
18. My faith in a Higher Power/Universal Intelligence helps me cope during challenges in my life.						
19. I strive to correct the excesses in my own lifestyle patterns/practices.						
20. I respect the diversity of people.						
21. Prayer is an integral part of my spiritual nature.						
22. At times, I feel at one with the universe.						
23. I often take time to assess my life choices as a way of living my spirituality.						
