# Supplementary material for: Coping as a Pathway Linking Religiosity and Spirituality to Mental Health and Early Cardio-Cerebrovascular Risk Among University Students in Malaysia
Source: Int J Environ Res Public Health. 2026 May 31;23(6):738. doi: 10.3390/ijerph23060738 (PMC13299289; doi:10.3390/ijerph23060738)
Supplement: Supplementary file 1 [file ijerph-23-00738-s001.zip › Supplementary File S5.pdf]

Supplementary File S5

The Spiritual Coping Questionnaire (SCQ)

1. Very Inaccurately
2. Rather Inaccurately
3. Neither inaccurately nor accurately
4. Rather Accurately
5. Very Accurately

	1	2	3	4	5
1. I was taking care of the environment.					
2. I was trying to find inner peace within myself					
3. I was accusing God/Higher Being for what happened in my life.					
4. I was trying to find relief in prayer.					
5. I tried to focus on the thought that God/Higher Being loves me.					
6. I was trying to find sense in what happened.					
7. I was seeking closeness to nature.					
8. I was convincing myself that other people were full of evil.					
9. I was trying to notice God's/Higher Being's presence in everyday life.					
10. I dreamed of ceasing to exist.					
11. I was taking care of other people.					
12. I was trying to prove to other people that they were egoists.					
13. I was turning to God/Higher Being with every matter important for me.					
14. I was trying to prove to other people that they were hypocritical.					
15. I was angry with God/Higher Being that He left me.					
16. I was seeking revenge on those who had hurt me.					
17. I was compassionate towards other people's pain.					
18. I was thinking that God/Higher Being punished me for my sins.					
19. I was convincing myself that I was a bad person.					
20. I was trying to find harmony with nature.					
21. I was convincing myself that my life had no goal whatsoever.					
22. I was trying to be fair towards other people.					
23. I was trying to notice the beauty and uniqueness of nature.					
24.I was convincing myself that my life had no sense.					
25. I was searching for strength to live in my relations with God/Higher Being.					
26. I was reacting when someone was hurt.					

27. I was trying to get to know myself better.					
28. I was trying to notice harmony in nature.					
29. I was nurturing my attitude of love toward other people.					
30. I was concentrating on the thought that God/Higher Being looked after me.					
31. I was trying to concentrate on my inner life.					
32. I was trying to help other people.					
